# 

**Published:** 1915-11

**Authors:** 


### Marriages.

Dr. C. C. Bradford of Godley, Texas, was recently married to Miss Mabel Hearn of Bremond.

Dr. Amos Hall Fortner of Dallas and Miss Martha Bryant of Whitewright were recently married.

Dr. Henry Beatty of Aransas Pass and Miss Jessie Richardson of Gregory were married in September.

### Deaths.

Dr. A. R. McCallon was recently shot to death by Hugh Donner at Cresson, Texas. Burial took place at his home near Greenville.

Dr. S. W. Smith, formerly of Denison, died recently in Port Huron, Mich.

Dr. H. Morrow of Caddo Mills died of heart failure at his home during September.

Dr. George W. Sims of San Antonio recently shot himself through the head, from which he died instantly.

Mrs. C. H. Beatty, wife of Dr. C. H. Beatty, a prominent physician at Aransas Pass, died recently.

Dr. G. W. Turner of Gainesville died at his home in that city the latter part of September.

